# A study predicting long-term survival capacity in postoperative advanced gastric cancer patients based on MAOA and subcutaneous muscle fat characteristics

**DOI:** 10.1186/s12957-024-03466-7

**Published:** 2024-07-16

**Authors:** Yubo Han, Yaoyuan Chang, Jiaqi Wang, Nanbo Li, Yang Yu, Zhengbo Yang, Weipeng Lv, Wenfei Liu, Jiajun Yin, Ju Wu

**Affiliations:** 1 Department of General Surgery, Affiliated Zhongshan Hospital of Dalian University, Dalian, China; 2 Department of Pathology, Affiliated Zhongshan Hospital of Dalian University, Dalian, China; 3 Department of Radiology, Affiliated Zhongshan Hospital of Dalian University, Dalian, China

**Keywords:** Gastric cancer, MAOA, Immunohistochemistry, Long-term survival, Predictive model, Muscle fat

## Abstract

**Background:**

The prognosis of advanced gastric cancer (AGC) is relatively poor, and long-term survival depends on timely intervention. Currently, predicting survival rates remains a hot topic. The application of radiomics and immunohistochemistry-related techniques in cancer research is increasingly widespread. However, their integration for predicting long-term survival in AGC patients has not been fully explored.

**Methods:**

We Collected 150 patients diagnosed with AGC at the Affiliated Zhongshan Hospital of Dalian University who underwent radical surgery between 2015 and 2019. Following strict inclusion and exclusion criteria, 90 patients were included in the analysis. We Collected postoperative pathological specimens from enrolled patients, analyzed the expression levels of MAOA using immunohistochemical techniques, and quantified these levels as the MAOAHScore. Obtained plain abdominal CT images from patients, delineated the region of interest at the L3 vertebral body level, and extracted radiomics features. Lasso Cox regression was used to select significant features to establish a radionics risk score, convert it into a categorical variable named risk, and use Cox regression to identify independent predictive factors for constructing a clinical prediction model. ROC, DCA, and calibration curves validated the model’s performance.

**Results:**

The enrolled patients had an average age of 65.71 years, including 70 males and 20 females. Multivariate Cox regression analysis revealed that risk (*P* = 0.001, HR = 3.303), MAOAHScore (*P* = 0.043, HR = 2.055), and TNM stage (*P* = 0.047, HR = 2.273) emerged as independent prognostic risk factors for 3-year overall survival (OS) and The Similar results were found in the analysis of 3-year disease-specific survival (DSS). The nomogram developed could predict 3-year OS and DSS rates, with areas under the ROC curve (AUCs) of 0.81 and 0.797, respectively. Joint calibration and decision curve analyses (DCA) confirmed the nomogram’s good predictive performance and clinical utility.

**Conclusion:**

Integrating immunohistochemistry and muscle fat features provides a more accurate prediction of long-term survival in gastric cancer patients. This study offers new perspectives and methods for a deeper understanding of survival prediction in AGC.

**Supplementary Information:**

The online version contains supplementary material available at 10.1186/s12957-024-03466-7.

## Introduction

Advanced Gastric Cancer (AGC) is one of the most common cancers globally [1], with the highest incidence in East Asia [2]. Despite continuous advancements in medical technology and treatment methods, the survival rate for AGC remains relatively low. Consequently, effectively predicting postoperative survival rates in AGC patients has become a focal point for clinicians and researchers. Monoamine oxidase A (MAOA) is a mitochondrial enzyme that degrades amine neurotransmitters such as serotonin (5-HT), adrenaline, and dopamine [3]. As a result, MAOA has been extensively studied in neurological diseases like Parkinson’s disease and depression [4]. However, in cancer-related research, the release of 5-HT has been associated with tumor progression, and 5-HT acts as a growth factor for prostate cancer (PCa) cells [5]. Furthermore, elevated MAOA expression is linked to high-grade PCa and contributes to a poorly differentiated phenotype [6–9]. Studies have also indicated that MAOA expression is significantly downregulated in gastric cancer tissue. It regulates glycolysis and cellular energy metabolism, promotes cancer cell apoptosis, and correlates with adverse patient outcomes. MAOA emerges as an independent prognostic factor for gastric cancer patients [10]. This discovery supports our current research efforts.

Radiomics is a promising non-invasive method that analyzes conventional medical images to extract quantifiable data revealing biological features of pathological processes at the microscopic level [11, 12]. Computed Tomography (CT) scans offer advantages like repeatability, standardization, and quantitative data extraction. They are indispensable for diagnosis and follow-up [13]. CT-based radiomics has been demonstrated to aid in predicting treatment responses and outcomes for various cancers [14–16]. Previous research has predominantly focused on analyzing lesion features, yielding significant results. Our study aims to investigate muscle and fat characteristics at the L3 vertebral body level in cancer patients, representing our innovative approach. Skeletal muscle depletion has long been considered an independent prognostic factor for cancer [17], distinct from body mass index (BMI). Muscle wasting is characterized by decreased muscle strength and reduced muscle quantity or quality, commonly observed in gastric cancer patients [18]. Conversely, the prognostic impact of obesity (subcutaneous and visceral fat tissue) remains uncertain. Some studies suggest that overweight/obesity serves as a protective factor for survival in advanced cancer patients, exemplified by the “obesity paradox” in advanced gastric cancer [19]. Both muscle wasting and obesity can be accurately identified using CT, a recommended approach in guidelines [20].

Increasing evidence suggests that muscle and fat tissues play crucial roles in immune responses and are closely associated with cancer prognosis [21]. However, the role of body composition (muscle and fat) parameters and MAOA in predicting prognosis for advanced gastric cancer (AGC) patients remains unknown. Our study, which combines muscle and fat features with immunohistochemistry results and patients’ clinical baseline data, establishes a nomogram that accurately predicts long-term survival in AGC patients. Additionally, our research provides new insights into cancer prognosis assessment.

## Methods

### Patient selection and data collection

**Fig. 1:**
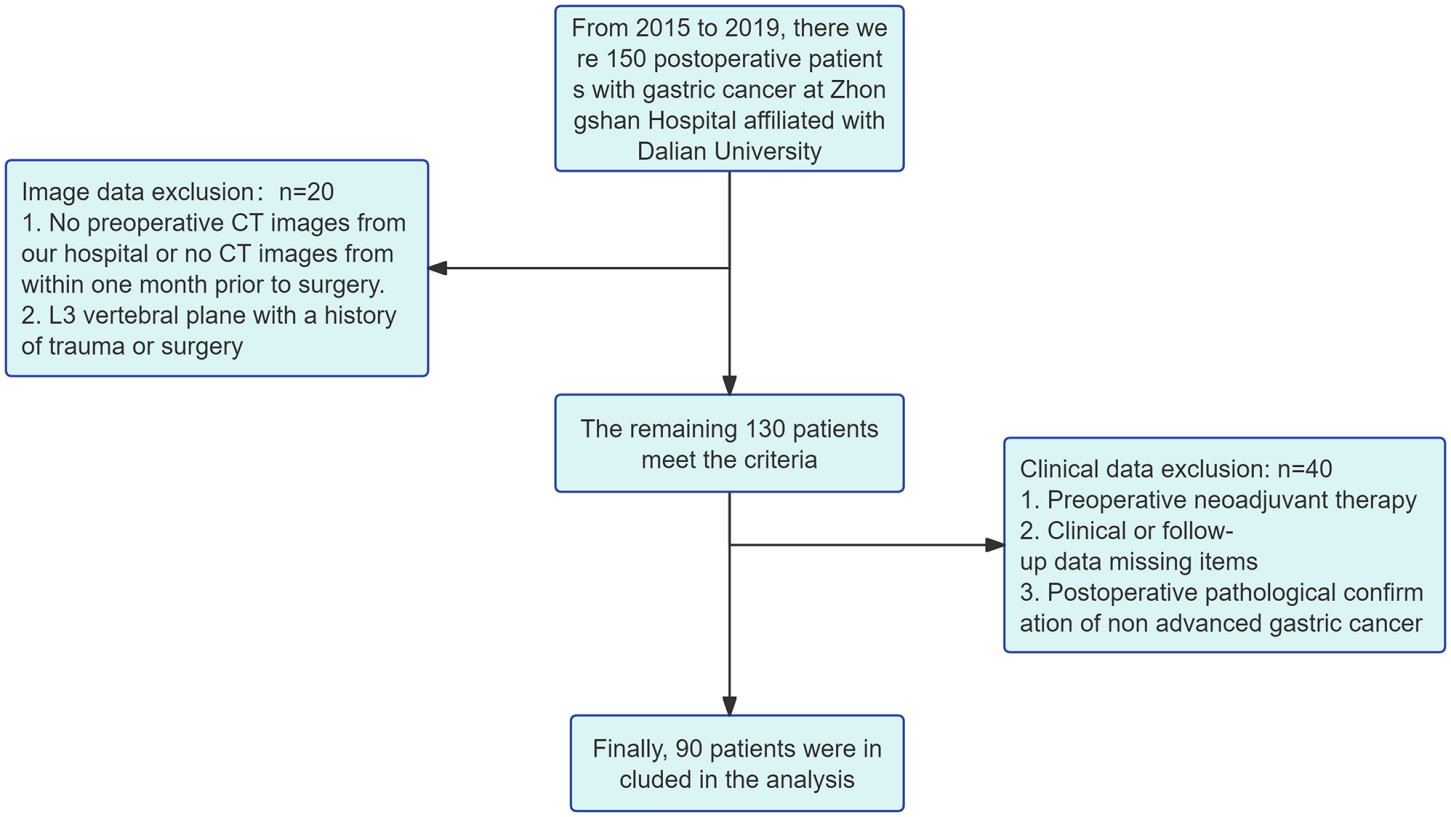
The flow diagram of patient selection

We conducted a retrospective analysis of data from advanced gastric cancer (AGC) patients who underwent curative surgery at Dalian University Affiliated Zhongshan Hospital between 2015 and 2019. The inclusion criteria were: (1) Patients who did not receive preoperative radiotherapy or chemotherapy. (2) Tissue confirmed by pathology as advanced gastric cancer after surgical resection. (3) Availability of complete clinical data and follow-up information. (4) No history of trauma or surgery at the L3 vertebral body level. (5) Postoperative patients who received guideline-recommended standard chemotherapy with sufficient cycles. (6) Availability of abdominal plain CT scan images taken within one month before surgery at our hospital. (7) Tumor stage II or III patients according to the 8th edition of the American Joint Committee on Cancer (AJCC) staging system. Exclusion criteria: Patients not meeting the inclusion criteria were excluded. Ultimately, out of 150 patients diagnosed with gastric cancer who underwent curative surgery, 90 patients were included in the analysis (Fig. 1). We analyzed the study subjects’ baseline, CT images, histopathology, and laboratory data. Postoperative follow-up occurred every three months during the first two years and every six months after that. The last follow-up date was October 31, 2023. Overall survival (OS) was defined as the duration from the surgery to the final follow-up or death date and served as the primary endpoint. Disease-specific survival (DSS) was defined as the time from the surgery date to all-cause death and served as the secondary endpoint.

### Immunohistochemical analysis of maoa expression level

**Fig. 2:**
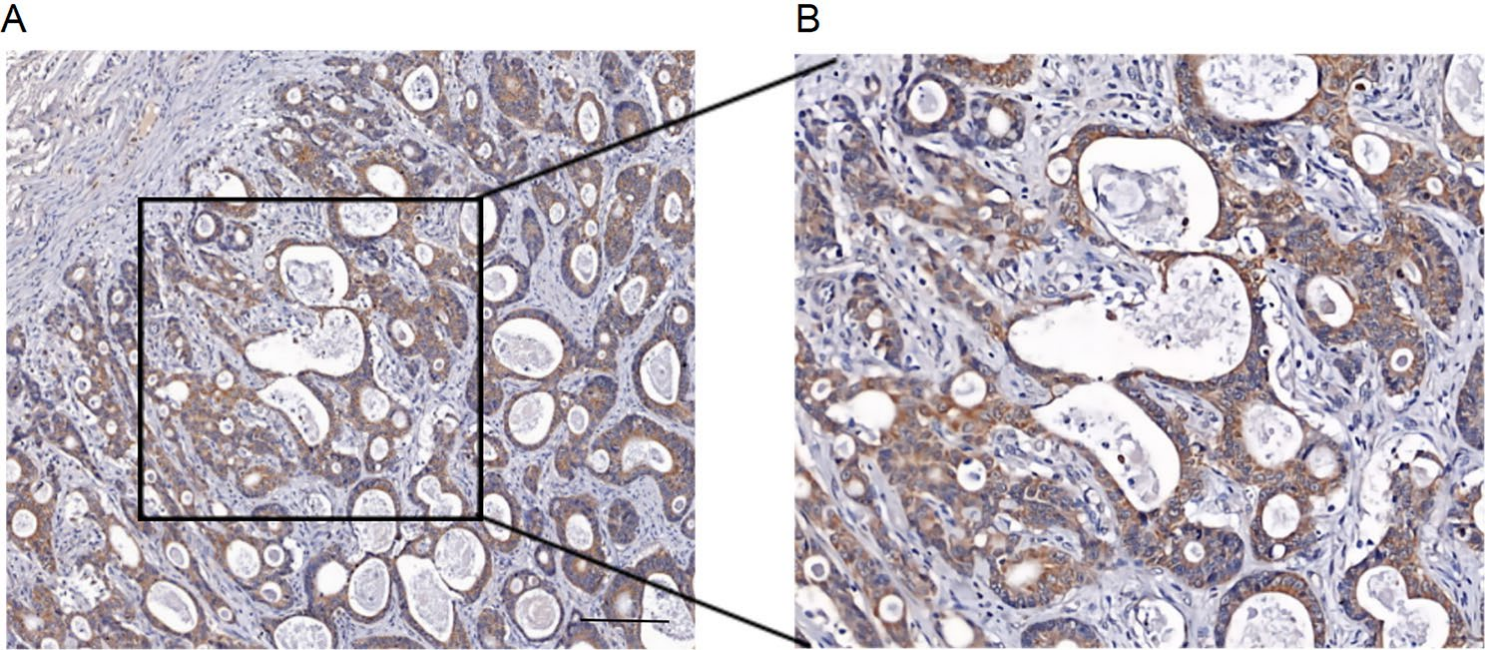
Quantitative IHC analysis of MAOA protein expression in AGC, Scale bars, 50μm

The tissue was fixed in 4% formalin buffer and subsequently embedded in paraffin. The tissue microarray (TMA) manufacturing process has been previously described in detail [22, 23]. In brief, one tissue spot (diameter: 0.6 mm) from each patient was transferred from a donor block containing cancer to an empty recipient paraffin block, creating tissue core blocks. These tissue core blocks were then sectioned, and the sections were transferred onto glass slides to generate tissue microarrays. After deparaffinization and rehydration in graded alcohols, the sections were incubated overnight at four °C with a primary antibody against MAO-A (diluted 1:100, BS6658, Bioworld Technology, Inc.). Subsequently, secondary antibody treatment was performed at room temperature. The sections were treated with an ammonia solution for 3 min and stained with hematoxylin for nuclear staining. Two experienced pathologists performed immunostaining (Fig. 2).

The completed chips were scanned and digitized using the fully automated digital slide system QuantCenter (3DHISTECH Ltd, Hungary). This system enabled routine quantitative analysis of bright-field and fluorescence images. It intelligently separated tumor areas stained by immunohistochemistry, allowing for quantitative analysis of immunohistochemical cell nuclei, cell membranes, and cytoplasm. Finally, the expression results of MAOA were transformed into a continuous.

variable called MAOAHscore. Using the “survival” package in R, a cutoff value of 20.40 was determined. Based on this cutoff value, patients were divided into two groups: those with MAOAHscore ≤ 20.40 were classified as the MAOAHscore = 0 group, and those with MAOAHscore > 20.40 were classified as the MAOAHscore = 1 group.

### Processing of abdominal CT images and radiomics feature extraction

**Fig. 3:**
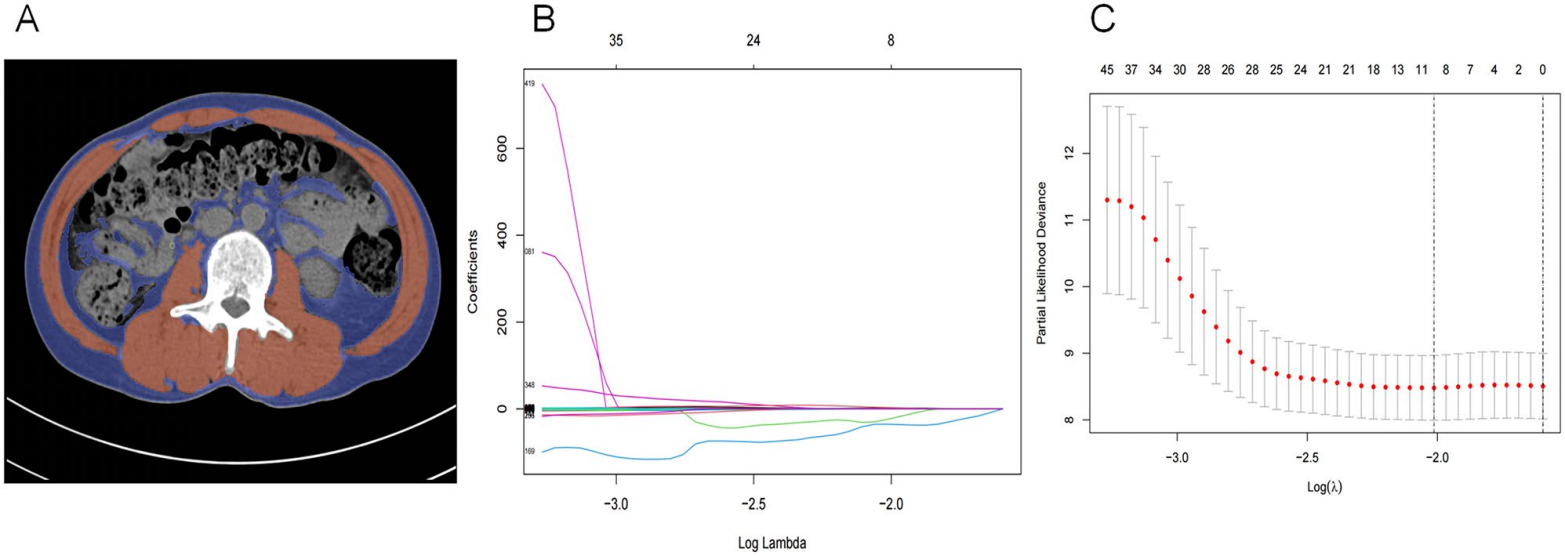
ROIs delineation and feature screening, (**A**),image after delineation by 3DSlicer, blue represents subcutaneous fat and viscerel fat, red represents muscles in L3 plane. (**B**),(**C**) Feature extraction and screening

We obtained preoperative abdominal CT images for these 90 patients from the hospital’s image repository. Using 3DSlicer [24], we delineated regions of interest (ROI) and performed Python-based feature extraction. Here are the specifics: The CT scanner was the Siemens SOMATOM Definition Edge CT (Approval Number: National food and drug administration equipment (into) the word,20,173,301,120), with a standard slice thickness of 5.0 mm. The 3DSlicer software was used to identify and quantify muscle and fat tissues [25]. By setting density ranges representing the ROIs, specific tissues (such as muscle or fat) could be selectively visualized. We selected a density range of -29 to + 150 Hounsfield units (HU) based on existing literature. Subsequently, we performed a more detailed segmentation of the ROIs (skeletal muscle and fat tissue) at the L3 level (Fig. 3A). An experienced radiologist with over ten years of experience did the entire segmentation process.

Using the Python package PyRadiomics, we batch-extracted features from the ROIs and saved them to an Excel file. In total, 1335 CT radiomics features were extracted. Feature selection and model risk scoring were performed using Lasso COX (ten-fold cross-validation) (Fig. 3B, C). The names of the features selected after filtration are shown in Supplementary Table 1. We used the median value of 92.95 as the cutoff to divide patients into two groups: those with risk ≤ 92.95 were classified as the risk = 0 group, and those with risk > 92.95 were classified as the risk = 1 group.

**Table 1:** Data distribution of different groups within 3-year OS

Variable	risk = 0	risk = 1	*P*-value	MAOAHScore = 0	MAOAHScore = 1	*P*-value
	(*N* = 45)	(*N* = 45)		(*N* = 40)	(*N* = 50)	
**Age**			0.966			0.211
≤ 65	23 (51.1%)	23 (51.1%)		17 (42.5%)	29 (58.0%)	
> 65	22 (48.9%)	22 (48.9%)		23 (57.5%)	21 (42.0%)	
**Sex**			0.205			0.958
Female	13 (28.9%)	7 (15.6%)		9 (22.5%)	11 (22.0%)	
male	32 (71.1%)	38 (84.4%)		31 (77.5%)	39 (78.0%)	
**Smoke**			0.668			0.402
No	28 (62.2%)	25 (55.6%)		26 (65.0%)	27 (54.0%)	
Yes	17 (37.8%)	20 (44.4%)		14 (35.0%)	23 (46.0%)	
**Drink**			0.933			0.155
No	34 (75.6%)	35 (77.8%)		34 (85.0%)	35 (70.0%)	
Yes	11 (24.4%)	10 (22.2%)		6 (15.0%)	15 (30.0%)	
**BMI**			0.281			0.976
≤ 25	34 (75.6%)	39 (86.7%)		33 (82.5%)	40 (80.0%)	
> 25	11 (24.4%)	6 (13.3%)		7 (17.5%)	10 (20.0%)	
**Alb**			0.986			0.708
≤ 29.6	5 (11.1%)	5 (11.1%)		12 (30.0%)	18 (36.0%)	
> 29.6	40 (88.9%)	40 (88.9%)		28 (70.0%)	32 (64.0%)	
**Size**			0.816			0.336
≤ 4.5	14 (31.1%)	12 (26.7%)		9 (22.5%)	17 (34.0%)	
> 24.5	31 (68.9%)	33 (73.3%)		31 (77.5%)	33 (66.0%)	
**Grade**			0.65			0.097
M	14 (31.1%)	17 (37.8%)		18 (45.0%)	13 (26.0%)	
L	31 (68.9%)	28 (62.2%)		22 (55.0%)	37 (74.0%)	
**TNM**			0.168			0.817
II	17 (37.8%)	10 (22.2%)		13 (32.5%)	14 (28.0%)	
III	28 (62.2%)	35 (77.8%)		27 (67.5%)	36 (72.0%)	

### COX univariate and multivariate analysis and KM curve plotting

We collected clinical features of patients (such as age, gender, tumor stage, etc.) along with RS and MAOAHscore as predictive factors. COX univariate and multivariate regression analyses were conducted to identify independent prognostic risk factors. Using the Kaplan-Meier method, we plotted survival curves to evaluate survival differences between different groups further.

### Establishment and validation of the nomogram

Based on the results of the COX model, we combined patients’ clinical data, radiomics features, and immunohistochemistry results to establish a nomogram model using the Cox proportional hazards model. This model categorizes patients into risk groups based on predictive factors, visually representing their prognosis. Finally, we will evaluate the predictive performance of the nomogram model using Receiver Operating Characteristic ROC curves. Additionally, we will create Decision Curve Analysis (DCA) curves to assess the model’s practical utility in clinical decision-making.

### Data analysis

We analyzed all data using R version 4.2.3 (The R Foundation for Statistical Computing, Vienna, Austria; http://r-project.org). Categorical variables were assessed using chi-square tests or Fisher’s exact tests. Survival analysis was performed using the Kaplan-Meier (KM) method, and statistical comparisons were made using the log-rank test. Subsequently, we used LASSO Cox regression to establish a radionics risk score. Univariate and multivariate Cox models were employed to identify independent prognostic factors associated with 3-year overall survival (OS) and disease-specific survival (DSS). The area under the ROC curve (AUC) was used to compare the predictive abilities of different indicators. Calibration and DCA curves were used to evaluate the accuracy and clinical benefit of the model.

## Results

In this study, among the 90 eligible patients with a 3-year overall survival (OS) endpoint, 46 were aged 65 years or older, and 44 were younger than 65. In the risk = 0 group, there were 32 males and 13 females; in the risk = 1 group, there were 38 males and seven females. There were no statistically significant differences between the two groups regarding age, gender, body mass index, smoking history, alcohol history, albumin (ALb) levels in blood, tumor size, postoperative pathological TNM stage, or differentiation degree. Similarly, there were no significant statistical differences in the variables included between the MAOAHScore = 0 and MAOAHScore = 1 groups (Table 1). The 3-year disease-specific survival (DSS) data distribution for patients is presented in the supplementary Table 2.

**Fig. 4:**
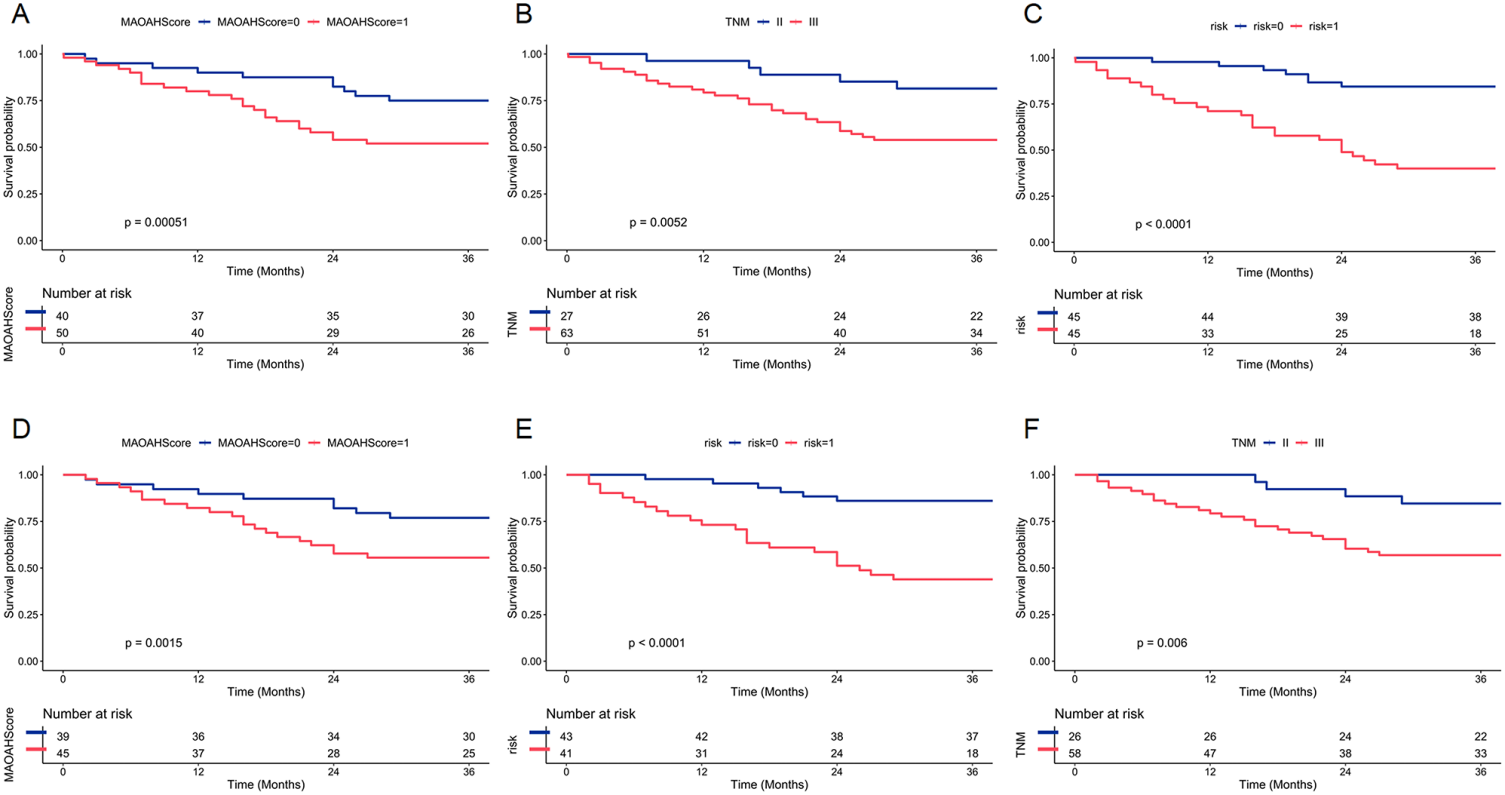
KM curve analysis. (**A**-**C**), KM curves analysis of each factor in 3-year OS.(**D**-**E**), KM curves analysis of each factor in 3-year DSS

When performing univariate COX analysis on the included variables, we found that risk (*P* < 0.001, HR = 4.753), MAOAHScore (*P* = 0.001, HR = 3.076), and TNM stage (*P* = 0.031, HR = 2.434) were significantly associated with 3-year OS and in the multivariate analysis incorporating the above three variables, risk (*P* = 0.001, HR = 3.303), MAOAHScore (*P* = 0.043, HR = 2.055), and TNM stage (*P* = 0.047, HR = 2.273) emerged as independent prognostic risk factors for 3-year OS (Table 2). The results of both univariate and multivariate COX regression analyses for 3-year DSS consistently demonstrate that risk, TNM stage, and MAOAHScore are independent prognostic factors (Supplementary Table 3). Survival analysis of independent prognostic factors revealed that Patients in the risk = 0 group had significantly higher 3-year OS than those in the risk = 1 group (*P* < 0.0001). Patients with TNM stage II had substantially higher 3-year OS than those with stage III (*P* = 0.0052). Patients in the MAOAHScore = 0 group had significantly higher 3-year OS than those in the MAOAHScore = 1 group (*P* = 0.00051) ( Fig. 4A-C). The survival analysis of 3-year DSS for each factor is shown in Fig. 4D-F.

**Table 2:** Univariate and multivariate Cox regression analysis for 3-year OS

	Univariate Analysis			Multivariate Analysis		
Variable	*P*-value	HR	95.0% CI	*P*-value	HR	95.0% CI
**Sex**	0.763	1.119	0.538–2.329			
**Age**	0.514	0.822	0.457–1.48			
**Smoke**	0.45	0.793	0.433–1.449			
**Drink**	0.272	0.65	0.301–1.402			
**BMI**	0.418	0.716	0.32–1.606			
**Size**	0.092	1.875	0.902–3.898			
**Alb**	0.966	0.987	0.529–1.842			
**Grade**	0.222	0.673	0.356–1.271			
**TNM**	0.031	2.434	1.086–5.459	0.047	2.273	1.01–5.115
**risk**	0.001	4.753	2.386–9.467	0.001	3.303	1.597–6.833
**MAOAHScore**	0.001	3.076	1.575–6.009	0.043	2.055	1.022–4.133

**Fig. 5:**
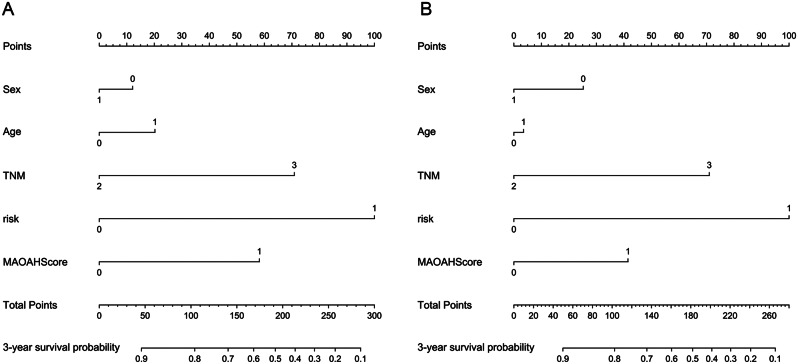
Nomogram predicts 3-year OS and DSS of patients (**A**), Nomogram predicts 3 (**B**), Nomogram predicts year OS

**Fig. 6:**
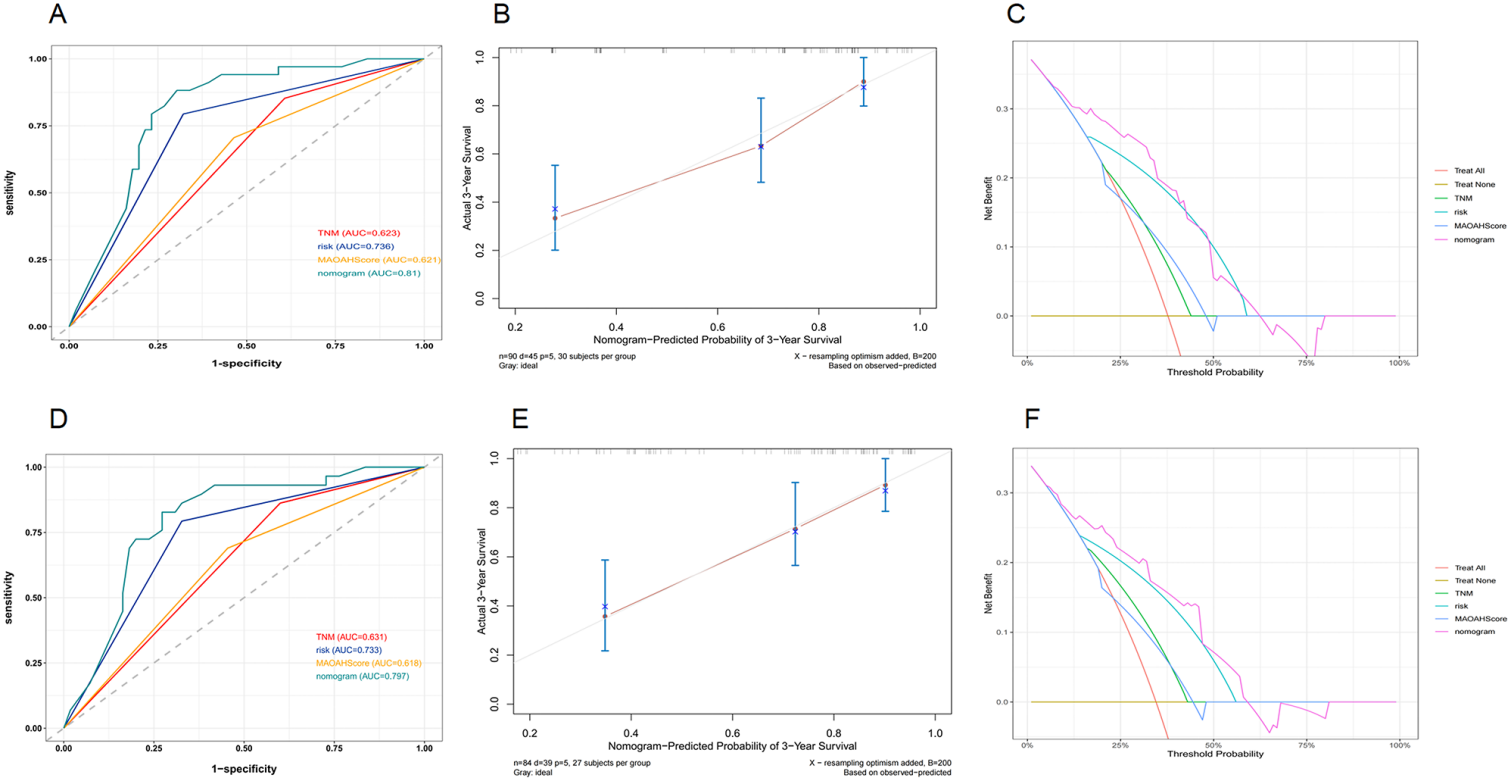
Model evaluation. (**A**-**C**),ROC, calibration,DCA curves to evaluate the nomogram to protect 3-year OS, (**D**-**F**), ROC, calibration, DCA curves to evaluate the nomogram to predict 3-years DSS

We further constructed nomograms for predicting 3-year OS (Fig. 5A) and 3-year DSS (Fig. 5B) by combining independent prognostic factors from COX proportional hazards regression analysis with clinical baseline data (gender and age). These nomograms assign scores to different groups based on various factors. The intersection of the cumulative scores of individual variables with the bottom scale represents the probability of death. The predictive performance of the nomogram was evaluated using Receiver Operating Characteristic (ROC) curves. The AUC values for predicting 3-year OS were as follows: nomogram (AUC = 0.81), TNM (AUC = 0.623), risk (AUC = 0.736), and MAOAHScore (AUC = 0.621) (Fig. 6A), The AUC of each indicator for predicting 3-year DSS is shown in Fig. 6D.

We plotted calibration curves for the nomogram to assess the consistency between predicted and observed 3-year OS (Fig. 6B) and DSS (Fig. 6E). A good alignment between the prediction and observation curves indicates favorable consistency. Finally, DCA demonstrated that using this predictive model for postoperative long-term survival can provide additional clinical benefits (Fig. 6C, F).

## Discussion

In this study, we accurately predicted the 3-year overall survival (OS) and disease-specific survival (DSS) of postoperative advanced gastric cancer (AGC) patients using a nomogram established by combining muscle and fat features with immunohistochemistry results. Previous research has rarely explored the joint analysis of muscle and fat features or the integration of immunohistochemistry in predictive models. We identified only two relevant articles concerning MAOA in gastric cancer. One study confirmed MAO-A’s involvement in mitochondrial dysfunction and aerobic glycolysis through phenotypic experiments, leading to the proliferation and metastasis of human gastric tumor cells [10]. However, this study did not delve into the molecular mechanisms of MAOA in gastric cancer development or consider its expression in gastric cancer tissues for clinical relevance. Another study mentioned that MAOA can interact with NDRG1, inhibiting downstream PI3K/AKT/mTOR pathway activity, thereby attenuating the Warburg effect in gastric cancer cells and ultimately suppressing tumor cell proliferation and malignant behavior [9]. These conflicting results warrant further investigation. Nevertheless, our center’s data analysis supports the viewpoint that elevated MAOA expression signifies a worse prognosis. Additionally, MAOA has been extensively studied in prostate cancer (PCa) and plays a critical role in nearly every stage, including castration-resistant prostate cancer, neuroendocrine prostate cancer, metastasis, drug resistance, stemness, and perineural invasion. Furthermore, MAOA up-regulation occurs not only in cancer cells but also in stromal cells, tumor-infiltrating T cells, and tumor-associated macrophages [26]. Elevated MAOA supports PC growth and progression by inducing stromal reprogramming and activating paracrine Twist1/IL-6/STAT3/CD44 signaling. IL-6 re-leased during this process has been shown to regulate the expression of almost all cancer biomarkers and various cancer-related signaling pathways [27]. While much remains to be explored regarding MAOA, existing evidence suggests its multifaceted role in cancer. Further research is needed to elucidate its specific functions in cancer pathogenesis.

Computerized tomography (CT) offers advantages such as repeatability, standardization, and quantitative data extraction. It plays an indispensable role in diagnosis and follow-up assessments. Currently, CT-based imaging remains the preferred standard for evaluating tumor drug responses in clinical trials. Radiomics based on CT has been shown to predict treatment responses and outcomes in various cancers, including colorectal cancer, gastric cancer [14, 28], and small-cell lung cancer [29, 30]. However, most studies focus on lesion-based analyses rather than intrinsic body composition parameters.

In our study, we innovatively selected the muscle and fat region at the L3 vertebral level as the region of interest (ROI). This choice maximally reflects patients’ nutritional status, as different nutritional statuses correspond to distinct imaging features. Evaluating muscle and fat feature parameters from two vertebral levels is a novel approach. Additionally, the combination of MAOA with muscle and fat features at the L3 vertebral level has rarely been explored. Fortunately, our results aligned with our expectations.

By assessing MAOA expression levels and muscle-fat features, we can more accurately predict long-term survival in gastric cancer patients and provide evidence for tailoring different treatment strategies. However, our study has limitations. Firstly, we included data from only one hospital due to design constraints, potentially introducing selection bias. Secondly, our research focused solely on the role of the MAOA gene and imaging features in predicting gastric cancer patients’ long-term survival without considering other potential biomarkers. Therefore, future research should expand sample sizes and incorporate additional bioinformatics and imaging techniques to evaluate cancer patients’ prognosis comprehensively.

Several avenues for future research are worth exploring. First, further investigations into the mechanisms of MAOA in cancer development and its interactions with other biomarkers and signaling pathways are warranted. Second, integrating more bioinformatics technologies, such as genomics, proteomics, and metabolomics, into prognostic assessments could establish more comprehensive predictive models. Large-scale, multicenter prospective studies are needed to validate our findings and explore their clinical applications. These endeavors may contribute significantly to personalized cancer treatment and prognosis assessment in clinical practice.

## Conclusion

This study established a comprehensive predictive model by integrating immunohistochemical analysis of MAOA expression and muscle-fat features at the L3 vertebral level. This model accurately predicts the long-term survival of patients with advanced gastric cancer (AGC) and holds clinical value. We can better identify high-risk patients and provide reliable evidence for personalized treatment strategies by emphasizing the importance of combining biomarkers and imaging features in clinical practice. Looking ahead, we anticipate further refining our comprehensive predictive model and enhancing its clinical applicability, ultimately contributing to improved prognosis assessment and treatment decisions for gastric cancer patients.

### Electronic supplementary material

Below is the link to the electronic supplementary material.

Supplementary Material 1

## Data Availability

No datasets were generated or analysed during the current study.
