# Supplementary material for: A study predicting long-term survival capacity in postoperative advanced gastric cancer patients based on MAOA and subcutaneous muscle fat characteristics
Source: World J Surg Oncol. 2024 Jul 16;22:184. doi: 10.1186/s12957-024-03466-7 (PMC11251287; doi:10.1186/s12957-024-03466-7)
Supplement: Supplementary file 1 — Supplementary Material 1 [file 12957_2024_3466_MOESM1_ESM.docx]

Supplementary Table 1. The names of the features selected after filtration

| **Feature name:** |
| --- |
| gradient_glszm_LowGrayLevelZoneEmphasis |
| gradient_glszm_SizeZoneNonUniformity |
| logarithm_glcm_Correlation |
| logarithm_glszm_LargeAreaLowGrayLevelEmphasis |
| wavelet-LHH_glrlm_LongRunLowGrayLevelEmphasis |
| wavelet-HHL_glszm_LargeAreaLowGrayLevelEmphasis |
| wavelet-HHH_glcm_DifferenceEntropy |
| wavelet-HHH_glcm_JointEntropy |

**Supplementary Table 2,** Data distribution of different groups within 3-year DSS.

| **Variable** | **risk=0** | **risk=1** | ***P*-value** | **MAOAHScore=0** | **MAOAHScore=0** | ***P*-value** |
| --- | --- | --- | --- | --- | --- | --- |
|  | **(N=43)** | **(N=41)** |  | **(N=33)** | **(N=51)** |  |
| **Age** |  |  | 0.887 |  |  | 0.065 |
| <=65 | 23 (53.5%) | 22 (53.7%) |  | 67.8 (8.72) | 63.8 (11.2) |  |
| >65 | 20 (46.5%) | 19 (46.3%) |  | 66.0 [54.0, 86.0] | 63.0 [37.0, 86.0] |  |
| **Sex** |  |  | 0.246 |  |  | 0.851 |
| FeMale | 13 (30.2%) | 7 (17.1%) |  | 7 (21.2%) | 13 (25.5%) |  |
| male | 30 (69.8%) | 34 (82.9%) |  | 26 (78.8%) | 38 (74.5%) |  |
| **Smoke** |  |  | 0.854 |  |  | 0.571 |
| No | 26 (60.5%) | 23 (56.1%) |  | 21 (63.6%) | 28 (54.9%) |  |
| Yes | 17 (39.5%) | 18 (43.9%) |  | 12 (36.4%) | 23 (45.1%) |  |
| **Drink** |  |  | 0.686 |  |  | 0.294 |
| No | 32 (74.4%) | 33 (80.5%) |  | 28 (84.8%) | 37 (72.5%) |  |
| Yes | 11 (25.6%) | 8 (19.5%) |  | 5 (15.2%) | 14 (27.5%) |  |
| **BMI** |  |  | 0.172 |  |  | 0.922 |
| ≤25 | 33 (76.7%) | 37 (90.2%) |  | 36 (81.8%) | 34 (85.0%) |  |
| >25 | 10 (23.3%) | 4 (9.8%) |  | 8 (18.2%) | 6 (15.0%) |  |
| **Alb** |  |  | 0.985 |  |  | 0.596 |
| ≤35 | 14 (32.6%) | 13 (31.7%) |  | 9 (27.3%) | 18 (35.3%) |  |
| >35 | 29 (67.4%) | 28 (68.3%) |  | 24 (72.7%) | 33 (64.7%) |  |
| **Size** |  |  | 0.806 |  |  | 0.966 |
| ≤4.5 | 20 (46.5%) | 17 (41.5%) |  | 15 (45.5%) | 22 (43.1%) |  |
| >4.5 | 23 (53.5%) | 24 (58.5%) |  | 18 (54.5%) | 29 (56.9%) |  |
| **Grade** |  |  | 0.874 |  |  | 0.144 |
| M | 14 (32.6%) | 15 (36.6%) |  | 15 (45.5%) | 14 (27.5%) |  |
| L | 29 (67.4%) | 26 (63.4%) |  | 18 (54.5%) | 37 (72.5%) |  |
| **TNM** |  |  | 0.301 |  |  | 0.374 |
| II | 16 (37.2%) | 10 (24.4%) |  | 16 (36.4%) | 10 (25.0%) |  |
| III | 27 (62.8%) | 31 (75.6%) |  | 28 (63.6%) | 30 (75.0%) |  |
| **Status** |  |  | <0.001 |  |  | 0.087 |
| Alive | 33 (76.7%) | 12 (29.3%) |  | 22 (66.7%) | 23 (45.1%) |  |
| Dead | 10 (23.3%) | 29 (70.7%) |  | 11 (33.3%) | 28 (54.9%) |  |

**Supplementary Table 3,** Univariate and multivariate Cox regression analysis for 3-year OS.

|  | **Univariate Analysis** | | | | **Multivariate Analysis** | | | |
| --- | --- | --- | --- | --- | --- | --- | --- | --- |
| **Variable** | ***p*-value** | **HR** | **95.0% CI** | | ***p*-value** | **HR** | **95.0% CI** | |
| Sex | 0.951 | 0.977 | 0.462 | 2.064 |  |  |  |  |
| Age | 0.89 | 1.046 | 0.555 | 1.97 |  |  |  |  |
| Smoke | 0.472 | 0.789 | 0.413 | 1.505 |  |  |  |  |
| Drink | 0.168 | 0.54 | 0.225 | 1.296 |  |  |  |  |
| BMI | 0.168 | 0.482 | 0.171 | 1.36 |  |  |  |  |
| Size | 0.874 | 1.057 | 0.533 | 2.096 |  |  |  |  |
| Alb | 0.061 | 1.918 | 0.971 | 3.791 |  |  |  |  |
| Grade | 0.061 | 1.918 | 0.971 | 3.791 |  |  |  |  |
| risk | 0 | 0.192 | 0.09 | 0.409 | 0.002 | 3.663 | 1.639 | 8.187 |
| pTNM | 0.01 | 3.139 | 1.314 | 7.499 | 0.035 | 2.588 | 1.07 | 6.255 |
| MAOAHScore1 | 0.001 | 3.959 | 1.955 | 8.015 | 0.031 | 2.307 | 1.078 | 4.938 |
